# Human perception and machine vision reveal rich latent structure in human figure drawings

**DOI:** 10.3389/fpsyg.2023.1029808

**Published:** 2023-02-23

**Authors:** Clint A. Jensen, Dillanie Sumanthiran, Heather L. Kirkorian, Brittany G. Travers, Karl S. Rosengren, Timothy T. Rogers

**Affiliations:** ^1^Department of Psychology, University of Wisconsin–Madison, Madison, WI, United States; ^2^Department of Brain and Cognitive Science, University of Rochester, Rochester, NY, United States; ^3^Department of Psychology, University of Rochester, Rochester, NY, United States; ^4^Department of Human Development and Family Studies, University of Wisconsin–Madison, Madison, WI, United States; ^5^Occupational Therapy Program, Department of Kinesiology, Waisman Center, University of Wisconsin–Madison, Madison, WI, United States

**Keywords:** human figure drawing, convolutional neural networks, children’s drawings, child development, machine vision, VGG 19, Draw-A-Person, ontogeny

## Abstract

For over a hundred years, children’s drawings have been used to assess children’s intellectual, emotional, and physical development, characterizing children on the basis of intuitively derived checklists to identify the presence or absence of features within children’s drawings. The current study investigates whether contemporary data science tools, including deep neural network models of vision and crowd-based similarity ratings, can reveal latent structure in human figure drawings beyond that captured by checklists, and whether such structure can aid in understanding aspects of the child’s cognitive, perceptual, and motor competencies. We introduce three new metrics derived from innovations in machine vision and crowd-sourcing of human judgments and show that they capture a wealth of information about the participant beyond that expressed by standard measures, including age, gender, motor abilities, personal/social behaviors, and communicative skills. Machine-and human-derived metrics captured somewhat different aspects of structure across drawings, and each were independently useful for predicting some participant characteristics. For example, machine embeddings seemed sensitive to the magnitude of the drawing on the page and stroke density, while human-derived embeddings appeared sensitive to the overall shape and parts of a drawing. Both metrics, however, independently explained variation on some outcome measures. Machine embeddings explained more variation than human embeddings on all subscales of the Ages and Stages Questionnaire (a parent report of developmental milestones) and on measures of grip and pinch strength, while each metric accounted for unique variance in models predicting the participant’s gender. This research thus suggests that children’s drawings may provide a richer basis for characterizing aspects of cognitive, behavioral, and motor development than previously thought.

## Introduction

In 1883, the Italian art historian Corrado Ricci was driven to shelter from the rain during his return from a monastery in Bologna. As he waited for the storm to pass, he noticed an interesting pattern in the crude drawings appearing along the side of his shelter’s archway: the drawings closer to the ground appeared less “technical and logical,” and also less vulgar, than those higher up. To Ricci, the observation suggested that the human drive to create images may follow a regular developmental trajectory, and the first effort to understand what children’s drawings suggest about their mental functioning was born.

Since Ricci’s (1887) treatise, many other scientists have seen the potential of drawings to evaluate children’s development due to the relatively consistent pattern of drawing progression found in typically developing children, as well as the unique characteristics of drawings produced by particular groups of children who were not neurotypically developing (Piaget, 1956; Goodnow, 1977; Gardner, 1980; Karmiloff-Smith, 1990; Cox, 1993; Case and Okamoto, 1996). For example, drawings have been used to assess children’s general developmental level (e.g., Denver Developmental Screening Test, [DDST]; Frankenburg and Dodds, 1967; McCarthy Scales of Children’s Abilities [MSCA]; McCarthy, 1972), children’s emotional functioning (e.g., kinetic family drawing; Koppitz, 1968; Burn and Kaufman, 1970; Naglieri et al., 1991), gender stereotypes in science (Miller et al., 2018), perceptual motor development (Bruininks-Oseretsky Test of Motor Performance II [BOT-II]; Bruininks and Bruininks, 2005), cognitive development (Piaget, 1956; Case and Okamoto, 1996), spatial reasoning (Freeman, 1980; Cox, 1986; Lange-Küttner and Ebersbach, 2013), and intellectual functioning (Goodenough, 1926; Harris, 1963; Koppitz, 1968; Naglieri, 1988; Arden et al., 2014). Drawings are also commonly used as part of neuropsychological assessments with adults, with the assumption that they provide a valuable source of evidence of cognitive and perceptual-motor abilities or impairments (Lezak, 1995; Smith, 2009). While researchers have used a variety of different drawing tasks, many of these assessments rely on human figure drawing, which is the task that we focus on in the current study.

Human figure drawings were initially used to provide a quick, initial evaluation of intelligence (e.g., Draw-A-Man; Goodenough, 1926; Goodenough-Harris Drawing Test; Harris, 1963; Draw-A-Child; McCarthy, 1972; Draw-A-Person; Naglieri, 1988). Such assessments evaluate the presence of important characteristics in drawings of human figures (e.g., body parts, facial features, body proportions) *via* a checklist. While simple to use, these coding scales fail to capture the rich structure apparent in children’s drawings, which potentially reflect perceptual, cognitive, and motor characteristics of the participant. In this study we describe two novel computational approaches to capturing the underlying structure in human figure drawings, then empirically assess whether the resulting descriptors can be used to predict individual cognitive, motor, and demographic characteristics of the participant.

**Figure 1:**
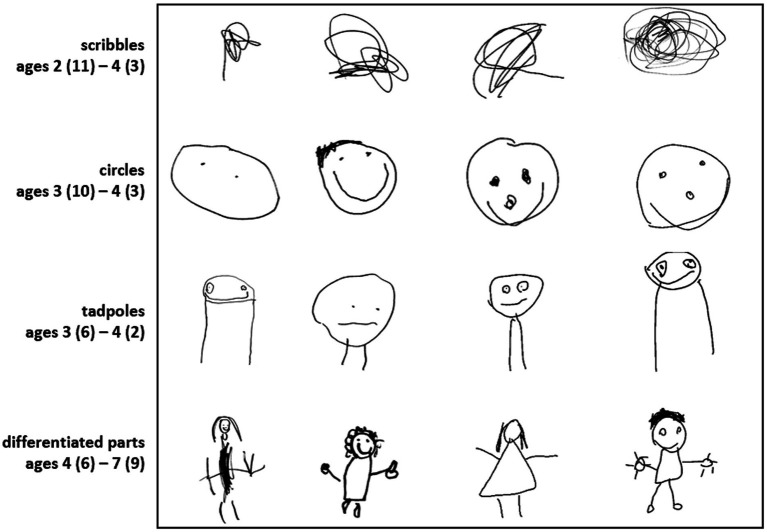
Patterns in human figure drawings across development. Examples of images produced in the drawing-across-media dataset, showing four common patterns previously identified in the literature.

Before describing our approach, it is useful to consider why human figure drawings have so long been viewed as providing insight into children’s mental abilities. Perhaps the clearest reason is that noted in Ricci’s original work: drawings produced by young children, though clearly simpler and less polished than those of older children and adults, are not arbitrary or random but exhibit common features across different ages and developmental stages (Kellogg, 1969; Cox, 1993). When drawing a person, scribbles transition to circles, then “tadpole” figures in which limbs are directly attached to a circular head. Older children differentiate the head from the body, gradually depict articulated limbs, and so on (Figure 1). It’s easy to see a parallel between the developing mind and these systematic changes in how children depict others, an observation that spurred the use of drawings to measure intelligence in childhood (Goodenough, 1926).

In addition to these patterns, drawings are useful for assessment because they possess an ecological validity, a generalizability to a child’s real life, uncharacteristic of most contemporary tools. Almost all children draw for fun. Unlike made-in-the-lab tools for measuring working memory, inhibition, or speed of processing (Weintraub et al., 2013; Zelazo et al., 2013), children performing a human figure drawing assessment will have had prior experience with the task, will not struggle to understand what is required, or to remember instructions, and will not typically find the task boring or unmotivating. Moreover, while most developmental assessments generate transient responses that the evaluator must score or transcribe, a drawing represents a permanent unfiltered record of the child’s behavior in the image produced. Another strength is that drawing relies minimally on language and so has the potential to measure aspects of cognition and behavior independent of linguistic capabilities. Perhaps most importantly, where many assessments seek to isolate and measure distinct, individual aspects of functioning, drawing requires the joint use and coordination of many faculties together: perception, imagery, spatial cognition, planning, conceptual knowledge, and motor control. Drawings thus have the potential to uncover many different and intersecting facets of the developing mind using an engaging task that does not rely heavily on language and that children regularly undertake in everyday life.

The central challenge for meeting this potential has been to develop a means of measuring the important underlying structure in the drawings children produce, and figuring out how to relate this to characteristics of the child (Beltzung et al., 2021; Sueur et al., 2021). Drawing is open-ended: a sketcher can depict even highly familiar and well-structured items like human figures in a bewilderingly large variety of ways. It is not immediately obvious which properties of children’s drawings “matter” for evaluating various mental or behavioral characteristics, or when the idiosyncrasies of their artwork reflect a mere flight of fancy versus a telling detail.

The earliest effort to formalize measurement of structure in drawings took the form of a detailed checklist and set of instructions for scoring. In the early 20^th^ century, Florence Goodenough used her experience with thousands of children’s human figure drawings to identify characteristics that, in her view, varied in a reliable manner across development. The original Draw-A-Man test (Goodenough, 1926) contained 46 standard features, with 5 additional items for images in profile, that should appear in the best drawings. An overall score was derived by raters inspecting a given drawing and checking off all the properties they could discern.

Subsequent decades saw both revisions and expansions to this general approach. Harris (Harris, 1963) expanded the checklist to include 71 features for drawings of a woman and 73 features for drawings of a man, and required children to draw a man, a woman, and ‘the self’ (p. 72). These categories were later adopted by Naglieri (1988), who again revised the checklist to include 65 features. Both tests developed quite stringent instructions for determining which depictions should receive full credit.

Other variants have sought to capture important structure more efficiently. Within the McCarthy Scales of Children’s Abilities (MSCA; McCarthy, 1972), for instance, the Draw-A-Child task requires only one human figure drawing, with the gender of the figure adjusted to match that of the child. The accompanying checklist includes just 10 items with possible scores of 2, 1, or 0 for each, yielding a maximum possible score of 20 points. The central aim was to measure non-verbal abilities within a battery of tasks that would be quick to administer and score for a practitioner. Despite its simplicity, McCarthy’s Draw-A-Child measure is highly correlated with both the longer Goodenough-Harris drawing test, *r* = 0.89 and the Full-Scale IQ measure of the Wechsler Intelligence Scales for Children, *r* = 0.68 (WISC-R; Wechsler, 1974; Naglieri and Maxwell, 1981). More recently, a variant of McCarthy’s system using a 12-point checklist (head, eyes, nose, mouth, ears, hair, body, arms, legs, hands, feet, clothes; see Arden et al., 2014) has been incorporated within a broad set of assessment tools used by the Twins’ Early Development Study (TEDS)—a large-scale longitudinal study of twins born in the United Kingdom between 1994 and 1996 and assessed at 2, 3, 4, 7, 9, 10, 12, 14, 16, 18, and 21 years of age (Saudino et al., 1998; Oliver and Plomin, 2007; Rimfeld et al., 2019). Researchers working on this study found that Draw-A-Child scores on the 12-point checklist taken at age 4 predicted a remarkable amount of variation in standard general IQ (*g*) measured in the same participants 10 years later (*r* = 0.20; Arden et al., 2014).

**Figure 2:**
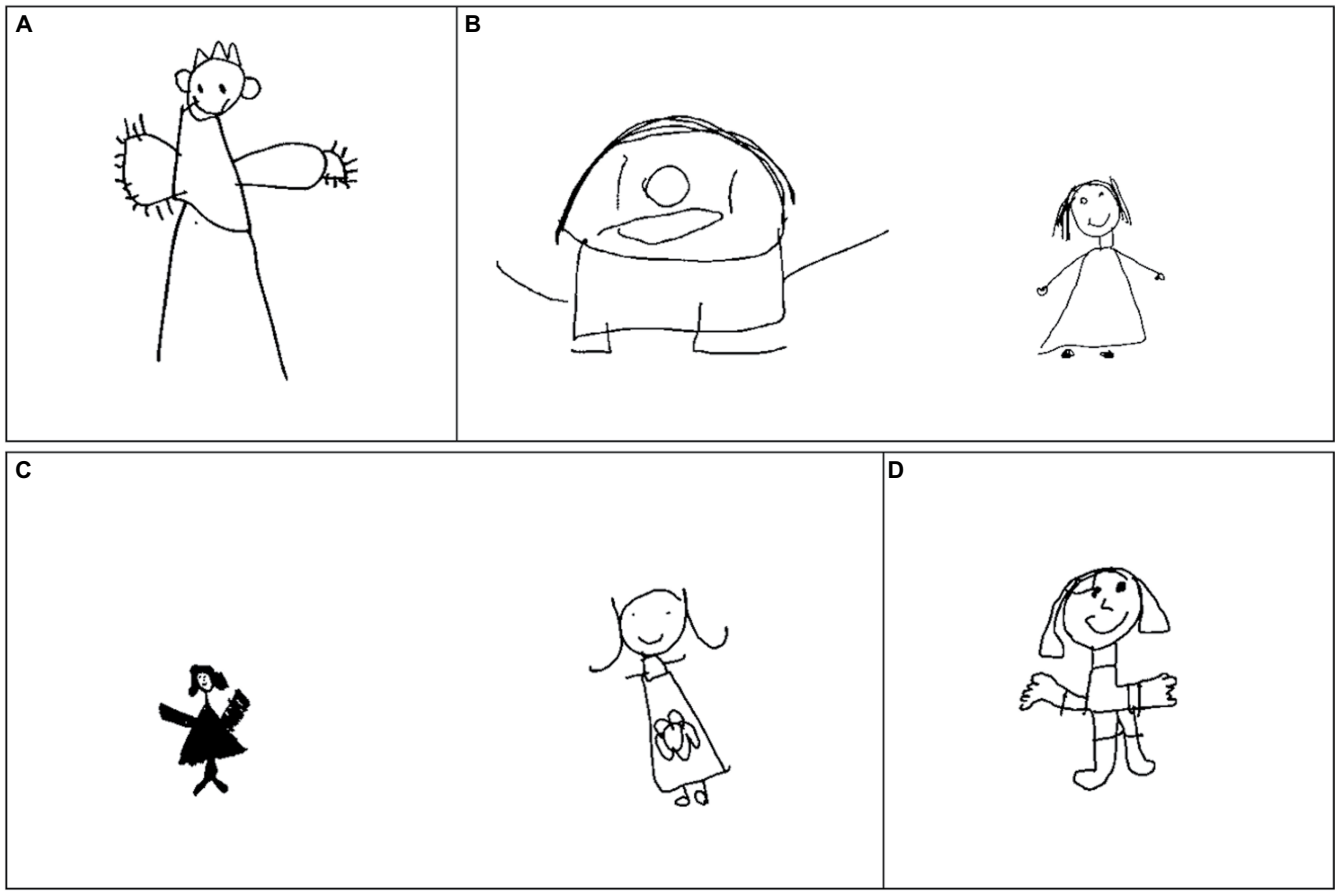
Limitations of the Draw-A-Child 12-item checklist. Examples of children’s drawings that demonstrate the limitations of the Draw-A-Child 12-item checklist. **(A)** Figure with lines that may suggest fingers extending from shapes that may constitute hands, but the Draw-A-Child checklist includes no way to indicate that there are too many fingers; **(B)** Two figures that each score a 9 on the Draw-A-Child checklist where the participant’s ability appears unequal; **(C)** Two figures that suggest different levels of pressure applied when making drawing; **(D)** Figure that presents with out of proportion features.

Yet for anyone who has skimmed through the various collections of children’s drawings that have accumulated over the years (Kellogg, 1969; Goodnow, 1977; Cox, 1993), it is clear that they possess more interesting structure than can be captured by checklists. Figure 2 shows several examples. In scoring the figure in panel A, the rater must decide whether it has fingers. Are the lines radiating out of each hand fingers, and if so, how does the rater indicate that there are more than five per hand? In panel B, both figures receive the same Draw-A-Child checklist score, but one is subjectively more accomplished than the other. In panel C, the drawings possess similar parts, but one has been rendered in much darker strokes than the other, indicating greater pressure on the writing implement that might in turn relate to the participant’s motor control. In panel D, the head is out of proportion to the body, which itself is out of balance, potentially reflecting difficulty in spatial reasoning or planning. Where checklists reduce the information in a drawing to a single number, in fact the latent information it contains may be multi-factorial and richer than pre-determined feature checklists can characterize (Beltzung et al., 2021; Sueur et al., 2021).

The central question we ask in the current work is whether new computational methods can improve on checklist-based measures by finding latent structure in children’s human figure drawings that relates reliably to their cognitive, motor, and demographic characteristics. The approaches we develop rely on two different innovations from recent years: (1) deep neural-network image classifiers, which learn complex features for representing visual images including sketches, and (2) techniques for exploiting human perception to embed images in low-dimensional spaces that reflect their overall perceptual similarity. Because these approaches are novel and their use as potential diagnostic tools has not previously been explored, the next section of the paper lays out each in detail. The following section then applies each to the analysis of human figure drawings produced by children and adults, evaluating whether the latent structure the new approaches uncover relates systematically to demographic, cognitive, and motor characteristics of the participants. The general discussion then considers what these results imply about the potential for more extensive use of children’s drawings in measuring aspects of cognitive, motor, and behavioral development.

## Section 1: Two novel techniques for measuring structure in drawings

### Approach 1: Machine-derived latent feature vectors

The first approach uses deep convolutional image classifiers to find latent structure in drawings. Such classifiers are neural network models that take bitmap images of objects as input and output an estimate of the semantic category to which the object belongs. Models of this type now routinely show human-level performance at categorizing color photographs of objects (Krizhevsky et al., 2012; Yu et al., 2022), and in learning to do so they acquire a complex set of *latent features* useful for representing the visual structure of objects. These representations are remarkably effective: though models are typically trained only on color photographs of real objects, the features they acquire capture the visual similarity existing between human-produced sketches and photographs of a given item (Fan et al., 2018). Indeed, prior work has shown that the feature-vectors from drawings produced by children at different ages trace out a reliable pattern: as children mature, the network-generated features grow increasingly similar both to those generated from adult drawings and those generated by photographs of corresponding objects (Long et al., 2018).

Inspired by this work, we used a well-known convolutional image classifier to extract visual features of sketches for use in cognitive/behavioral assessment. A full exegesis of convolutional neural networks is beyond the scope of this paper (see Kriegeskorte, 2015; Battleday et al., 2021; Li et al., 2022; for detailed surveys), but we provide a brief overview here before explaining how we have used the model.

In convolutional image classifiers, each bitmap pixel is represented by three input units encoding, as real-valued numbers, the amounts of red, green, and blue characterizing the pixel’s color. The input bitmap is divided into multiple overlapping “patches,” similar to spatial receptive fields in visual neuroscience. The input units within each patch project to a bank of feature-detectors or *filters*, with the activation of each filter indicating how strongly the corresponding feature can be detected in the input patch. The same filters get applied to each input patch, so that every patch in the image is recoded as an activation pattern across the same set of filters. This general structure is then repeated several times, with each successive layer receiving inputs from a spatially contiguous patch of earlier units, encoding the presence of increasingly complex features within increasingly broad regions of the input. The deepest such convolutional layer then projects to one or more “flat” layers that discard spatial/topographic information about features. The deepest flat layer in turn projects to an output layer in which each unit corresponds to a single category label. Activations of output units are positive and constrained to sum to one, so the activation pattern across units can be viewed as a probability distribution over the various possible categories. The model’s “job” is to take an image of an object as input, pass it through all model layers, and generate output activations that correctly indicate the probability that the depicted object belongs to each possible category.

Critically, the features detected at each model layer, and the activation pattern generated across units in the “flat” layers, are not pre-specified. Instead they are learned through error backpropagation by training the model to correctly categorize photographs from very large corpora of labelled images. Such training allows convolutional networks to classify new photographs with remarkable accuracy, and to learn visual features at each convolutional layer that express the visual structure of natural images and resemble, in some respects, neural responses to visual stimuli measured in human and non-human primate brains (Cadieu et al., 2014; Yamins et al., 2014; Güçlu and van Gerven, 2015; Cichy et al., 2016; Bao et al., 2020; Storrs et al., 2020).

We used the well-known VGG-19 model, a fully trained neural-network from the Visual Geometry Group at Oxford (Simonyan and Zisserman, 2014). This model has 16 convolutional layers and 3 fully connected “flat” layers intervening between input and output. It was trained to assign each of ~14 M ImageNet images to one of 1,000 possible mutually-exclusive categories. We selected VGG-19 because prior work has shown that its penultimate layer captures important similarity relations amongst sketches of objects (Fan et al., 2018), and because it has been studied extensively in visual cognition and neuroscience (Jha et al., 2020). The approach we describe here can, however, be easily extended to other visual neural network models.

Our goal was to use VGG-19 to extract visual feature vectors characterizing the complex visual structure of a given drawing, and to then assess whether these features reliably predict cognitive, behavioral, and demographic characteristics of the participant. To this end we devised the following procedure. Each drawing in a dataset was scanned and converted to a black-and-white bitmap of the appropriate dimensions (i.e., those of the VGG-19 input layer). The bitmap was fed into the trained network, which computed activation patterns across all units in each model layer. Following Fan et al. (2018), we extracted the activation pattern across the penultimate model layer (i.e., the last hidden layer before the outputs), and took this as a vector-based representation of the drawing.

The resulting vectors are very high-dimensional, since the corresponding layer has 4,096 units. Rather than using these activation vectors directly, we instead applied matrix decomposition methods to reduce the dimension. After extracting the VGG-19 vectors for each of *k* drawings in a dataset, we computed the *cosine similarity* between each vector pair, yielding a symmetric *k* by *k* matrix indicating the degree to which pairs of drawings are represented similarly by the model. We then used classical multidimensional scaling to compute *d* coordinates for each image, such that the pairwise similarities between all images in the *d-*dimension space approximate as closely as possible those in the original matrix.

**Figure 3:**
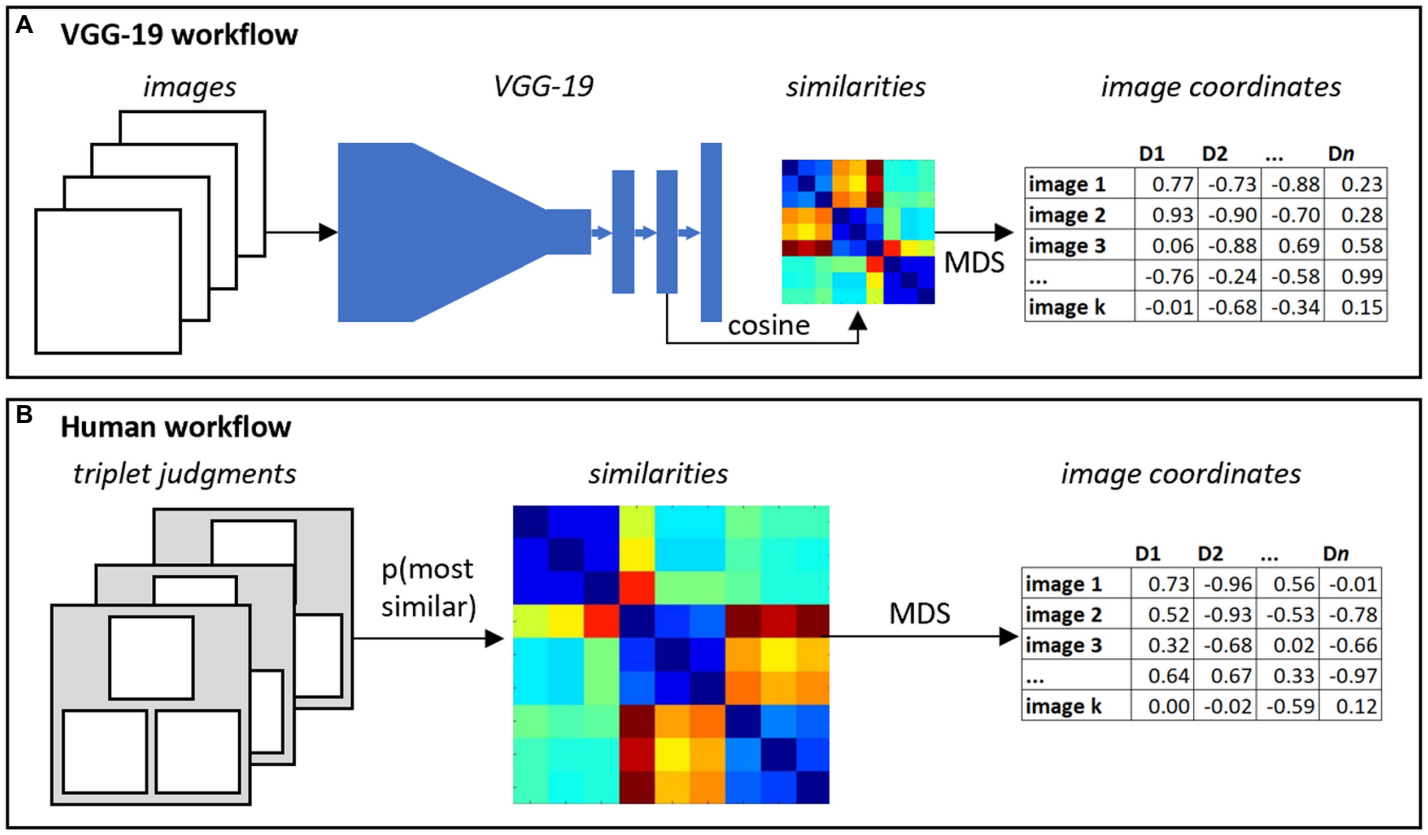
Two methods for capturing latent structure in sketches. **(A)** The VGG-19 workflow feeds each image into the neural network, extracts high-dimensional vectors from the penultimate layer, computes pairwise cosine similarities amongst all images, and reduces these to a small number of coordinates for each image using multidimensional scaling (MDS). **(B)** The human workflow collects most-similar judgments for a large set of triplets, computes pairwise probabilities that two images are chosen as most similar, and again uses MDS to reduce the similarity matrix to a small number of coordinates for each item.

The full procedure effectively re-represents each image as a *machine-derived latent feature vector* that captures similarities amongst VGG-19’s internal representations. The latent feature vectors can then be used in regression models to predict characteristics of the participant. The full workflow is shown in Figure 3A.

### Approach 2: Mining human perception to find structure in drawings

The second approach is motivated by the intuition that human perception of drawings can be sensitive to varieties of structure not captured by machine-vision techniques like VGG-19. For instance, people possess conceptual knowledge about items depicted in drawings; can easily decompose these into component parts; understand the structure and function of different drawing elements (for instance that limbs are jointed and can move around, or that hands can grasp); can interpret very simple features such as straight lines or circles as depicting more complex object parts like legs or heads; comprehend common drawing conventions such as the use of stick figures to represent the human form; and can easily evaluate overall quality of a drawing. All of this rich knowledge is absent in image classifiers and may inform the similarity judgments that people generate.

Prior work described in the introduction uses human raters to explicitly evaluate the presence of many pre-defined features in a drawing, a procedure that (a) requires expert knowledge of the checklist tool, (b) is laborious and time-consuming and (c) relies on the particular features chosen for inclusion on the checklist. Our approach instead makes use of the ability of non-experts to quickly and reliably judge the perceptual similarity and quality of drawings, in two related ways.

**Figure 4:**
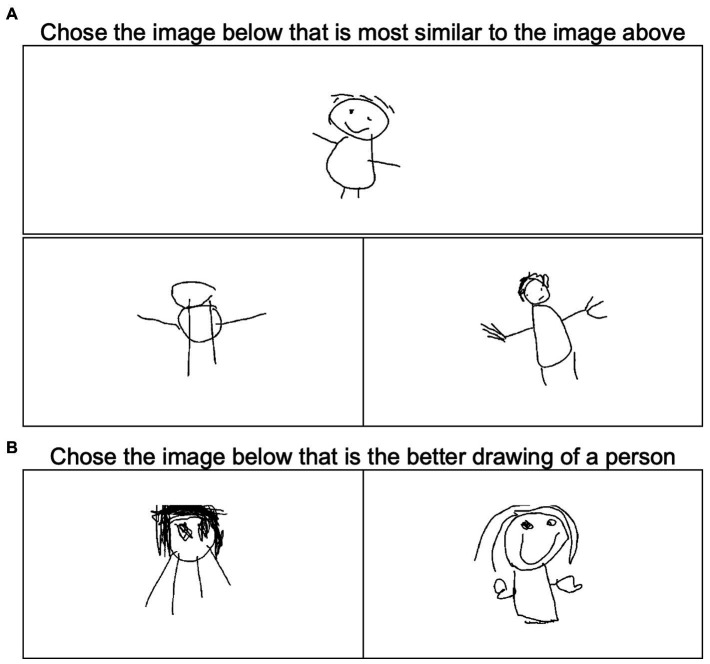
Examples of trials in human-judgment tasks. **(A)** One trial of the triadic judgement task where participants must decide which of the two bottom images is most perceptually similar to the top image. **(B)** One trial of the drawing quality judgment task, in which participants must decide which of two images is a better drawing of a person.

First, we employ a *triadic judgment task* to situate images within a low-dimensional space that expresses human perceived similarity (Jamieson et al., 2015). On each of many trials, human raters must judge which of two images is most perceptually similar to a third (Figure 4A). Judgments for triplets generated from a set of *k* images are collected online from many human workers (for instance, Amazon Mechanical Turk or [AMT] workers), and these are compiled to create a *k* by *k* matrix indicating, for any two items, the probability that they are selected as “most similar” relative to other images. In this way, we obtain discrete, forced-choice similarity judgements that can approximate continuous estimates of perceptual similarity. For the AMT worker, the task is simply to choose which of two images presented at the bottom of their screen is most similar to a third image presented at the top.

We then apply a multi-dimensional scaling algorithm suited to triplet judgements to embed the *k* items in a *d* dimensional space (Figure 3B). Specifically, we used the *crowd kernel* approach to ordinal embedding, which situates each drawing within an *n* dimensional space in such a way that items frequently selected as similar to one another across triplets are nearby (i.e., have low Euclidean distance) in the space. Just as with the VGG-19 workflow, this approach re-represents each image as a *human-derived latent feature vector*, with the similarity between vectors indicating the likelihood the two corresponding images are judged to be perceptually similar. As with VGG-19, these vectors can be used in regression to predict characteristics of the participant.

Second, we use a similar approach to evaluate the overall quality of a drawing as perceived by a non-expert human judge. On each trial a rater on AMT views two images depicting a human figure and must choose which is “the better drawing of a person” (Figure 4B). Many such judgments are collected from the crowd of AMT workers for random pairs of drawings, and for each we compute the proportion of trials for which a drawing was chosen as the best from among all trials where the image appeared. Using this forced-choice approach, high-quality drawings are those often chosen as the “best” compared to other images—that is, drawings selected on a high proportion of trials where they appear. Thus the “proportion selected” value provides an estimate of the true ranking of images by human-judged quality—we therefore refer to this metric as the *quality-rank score.* Like the checklist approach, this method produces a single number evaluating the perceived quality of the drawing, but unlike the standard method, it does not rely on presence/absence of pre-selected features, or any expert training or knowledge: the resulting measure instead reflects non-expert human judgements about the quality of each drawing.

Together the application of these methods to a set of drawings yields, for each image, (a) a machine-derived latent feature vector (b) a human-derived latent feature vector, and (c) a human-derived estimate of perceived drawing quality. These numeric descriptions of the images do not correspond to explicit, identifiable features of the kind appearing in checklists, but may capture underlying structure in drawings that nevertheless relate cognitive, behavioral, and demographic characteristics of the participant. The next section empirically tests this possibility.

## Section 2: An empirical proof-of-concept

We used these techniques to analyze a dataset recently collected as part of an unrelated project designed to understand how the introduction of touchscreen tablets into children’s homes might influence the quality of drawings they produce (Kirkorian et al., 2020). As part of the original study, the authors collected human figure drawings from 129 children aged 3–9 years and 29 young adults. Children completed an assessment of motor function (grip and pinch strength) and were additionally evaluated on the age-appropriate level of the 3^rd^ edition of the Ages and Stages Questionnaire (ASQ; Squires and Bricker, 2009), a parental-report based screening assessment that includes subscales for fine and gross motor control, problem solving, personality, and social behaviors. Parents also completed a demographic survey.

Our goal was to conduct a proof-of-concept analysis to determine whether the latent structure of drawings expressed by machine vision and/or human perception captures reliable information about the demographic, motor, or other characteristics of the participant as measured through the corresponding standard assessments. To this end, we applied each of the procedures previously described to generate coordinate vectors for each drawing from machine vision and human perception as well as human-judgment-based quality scores. We then used these metrics to predict the participant’s demographic characteristics (age and gender), motor capabilities, and other Ages and Stages subscores, focusing on three key questions:

1. Do the new metrics based on machine vision and/or human perception reliably predict variance in the outcome measures (participant age, gender, ASQ scores, etc.)?
2. Do metrics from machine vision and human perception account for similar or different characteristics of the participant?
3. Do the new metrics account for significant variance over and above the Draw-A-Child checklist score?

## Method

### Participants

The data come from prior studies of human-figure drawings conducted by several of the co-authors, approved by the Institutional Review Board for Education and Social/Behavioral Science at the University of Wisconsin-Madison (Protocol no. 2015–0564, “Children’s Drawing Across Media”). Data were collected between July and November 2015.

Children for these studies were recruited through preschools and a children’s museum in Madison, WI, a medium-sized city in the Upper Midwestern United States. Adults were recruited through personal contacts and snowball sampling from within the undergraduate population at UW-Madison. The sample included 129 children, ages are stated in year;month (age range = 1;10–8;10, *M* = 4;4, *SD* = 1;6, 53% female, 47% male) and 25 adults (age range = 19;1–22;0, *M* = 20;7, *SD* = 0;10, 76% female, 24% male). Parents of 85 children (66%) completed a brief demographic survey. The majority (*n =* 66, 77%) identified their child as White and non-Hispanic; other children were identified as Hispanic (*n =* 7, 8%), Asian/Pacific Islander (*n* = 5, 4%), Black/African American (*n* = 3, 2%), or other/mixed race (*n* = 4, 3%). For the parents, the mean years of education was 18;4 (*SD* = 2;10, range = 12–25), a level that is roughly equivalent to a master’s degree. Parents were also asked to place themselves on a Socioeconomic Status (SES) continuum derived from the MacArthur Scale of Subjective Social Status (Goodman et al., 2001). Respondents are asked to place themselves on a 10-point continuum with one anchor representing individuals who have the least money, least education, and either no job or a low-status job (rating of 1 out of 10) and the other anchor representing individuals who have high levels of money, high education, and a high-status job (rating of 10 out of 10). The average subjective SES for our sample was 7.15 (*SD* = 1.36, range = 4–10).

### Data collection procedure

#### Human figure drawings

Participants were prompted to draw a human figure following a script adapted from the Draw-A-Child protocol (McCarthy, 1972). Fifty-six children provided three drawings: one with marker on paper, one with finger on tablet, and one with stylus on tablet. Fifteen children contributed two drawings across the three media. The remaining fifty-seven children produced one drawing each, in one of the three media. Taken together, there were 255 drawings by children. The twenty-five adult participants each produced two drawings, one with marker on paper and one with finger on tablet, for a total of 50 drawings.

#### Grip and pinch strength

These measures were used to obtain an assessment of motor function. As the production of a drawing with an implement on a surface must compensate for frictional forces on the surface, both grip and pinch strength can be viewed as functional measures related to both gross and fine motor control. The full procedure for assessing grip and pinch strength can be found in Kirkorian et al. (2020). Briefly, a Preston Jamar hand dynamometer and pinch meter (Patterson Medical, Warrenville, IL) were used for these assessments. For grip strength, the smallest handle position was used for all participants. Participants were asked to attempt three assessments for each hand, alternating between both hands. The maximum grip and pinch measurements across all trials were used in the analyses, as recommended by Roberts et al. (2011).

#### Ages and stages questionnaire

Finally, parents of 45 children (56% of sample) completed the ASQ-3, a parent-report evaluation of developmental milestones across five domains: fine motor skill, gross motor skill, problem solving and personal/social skill. Parents completed the specific ASQ questionnaire that corresponded to their child’s chronological age in months by responding: Yes, Sometimes, or Not Yet, to questions about their child’s behaviors. For example, Gross Motor: “Does your child climb the rungs of a ladder of a playground slide and slide down without help?”; Fine Motor: “When drawing, does your child hold a pencil, crayon, or pen between her fingers and thumb like an adult does?.” For each domain, scores range from 0–60, with higher scores indicating increased developmental achievement.

### Image processing and rating procedure

#### Image pre-processing

Original drawings were produced with a black marker on white paper, or on a tablet computer using black script on a white background. Paper drawings were scanned, and screen shots were taken for tablet-based images. All drawings were digitized to a common format and cropped to remove identifying information (e.g., participant IDs) and unintended markings (e.g., borders, scanning artifacts) while maintaining the aspect ratio. The images were then contrast normalized so that all pixels were either black or white to ensure minimal low-level visual differences between scanned versions of paper images and drawings produced on tablets. All drawings were also centered and padded with white pixels to a uniform size.

#### Machine-derived latent feature vectors

All code for replicating our analyses is available at https://github.com/ClintJensen/DrawingsProject. We used a standard implementation of the VGG-19 architecture pre-trained to classify photographs of real objects within the ImageNet database (Deng et al., 2009; Simonyan and Zisserman, 2014). The model is coded in Python 3.6 using TensorFlow (1.13.1) libraries. Each pre-processed image was rescaled to the dimensions of the model input layer (3x224x224) and presented as input to the model. Activation patterns were computed at each layer in a feed-forward pass, and the resulting vectors from the penultimate layer for each image were extracted.

We next computed cosine similarities for all vector pairs, then decomposed the resulting matrix into a small number of components using classical multidimensional scaling. For purposes of data exploration and visualization, we computed embeddings in both two dimensions (each image represented with two coordinates) and five dimensions (each represented with five coordinates).

#### Human-derived latent feature vectors

Human-derived latent feature vectors were estimated from a large set of triplet judgments collected from 218 workers on Amazon Mechanical Turk (AMT). All workers had a HIT approval rating greater than 97%, completed a reCAPTCHA verification procedure before beginning, and worked from computers with IP addresses within the US.

Data were collected using NEXT, a software package that enables easy deployment of simple forced-choice experiments in the cloud (Jamieson et al., 2015). On each trial of the triadic judgments task, workers viewed a sample image above two option images and pressed the left or right arrow key to indicate which option was most similar to the sample (Figure 4A). Workers were asked to judge 200 randomly-selected triplets, a task designed to take 10 min given an average response time of 2.5 s per selection, but were permitted to exit at any time. For those exiting early, all data collected to that point were included in the analysis. For the triadic judgments task, out of our total 218 workers, the average number of selections made was 146 (51 workers completed all 200 image pairs). A total of 31,832 judgments were collected. AMT workers were paid $1.00 for participation.

From these data, 10% of trials were selected at random as hold-outs to evaluate the quality of embeddings. Embeddings were then estimated in 1–5 dimensions from the remaining data using Crowd Kernel, an algorithm designed specifically to learn non-metric embeddings from discrete comparative judgments of this kind (Tamuz et al., 2011). We computed the quality of each embedding by tabulating how often inter-item distances correctly predicted human decisions in the set of held-out triplets. Embeddings in 2-dimensions were found to have the best accuracy and were retained for the regression analyses. These same 2D embeddings were used within the visualizations that follow.

#### Drawing quality

Overall drawing quality was measured in two ways. First, we scored all drawings using the same 12-item Draw-A-Child checklist employed in the TEDS study described in the introduction (Saudino et al., 1998; Oliver and Plomin, 2007; Arden et al., 2014). Two trained raters independently scored all drawings, indicating which of the 12 features they detected in the image. This procedure yielded a total score from 0 to 12 for each image. Inter-rater reliability across drawings showed a by-item Pearson’s product–moment correlation of 0.93. The final score for each drawing was taken as the mean of the two raters.

The second approach used pairwise judgments to define each drawing’s perceived quality-rank score using the forced-choice method described earlier, again crowdsourced from AMT workers, using the same recruitment procedures and controls. On each trial of this procedure a worker saw two drawings and was asked to decide which was a better drawing of a person by pressing the left or right arrow key (Figure 4B). A total of 58 workers were asked to judge 200 pairs but were permitted to stop at any point. The average number of selections per participant was 174 (25 participants completed all 200 image pairs). As with the triadic judgements task, all data from all respondents were included in the analysis. A total of 10,107 judgments were collected. Each worker was paid $1.00. The quality score for each drawing was then computed as the number of times the image was chosen as the better drawing divided by the total number of times it appeared in the dataset, a proportional value ranging from 0 to 1.

## Results

### Exploratory analyses

**Figure 5:**
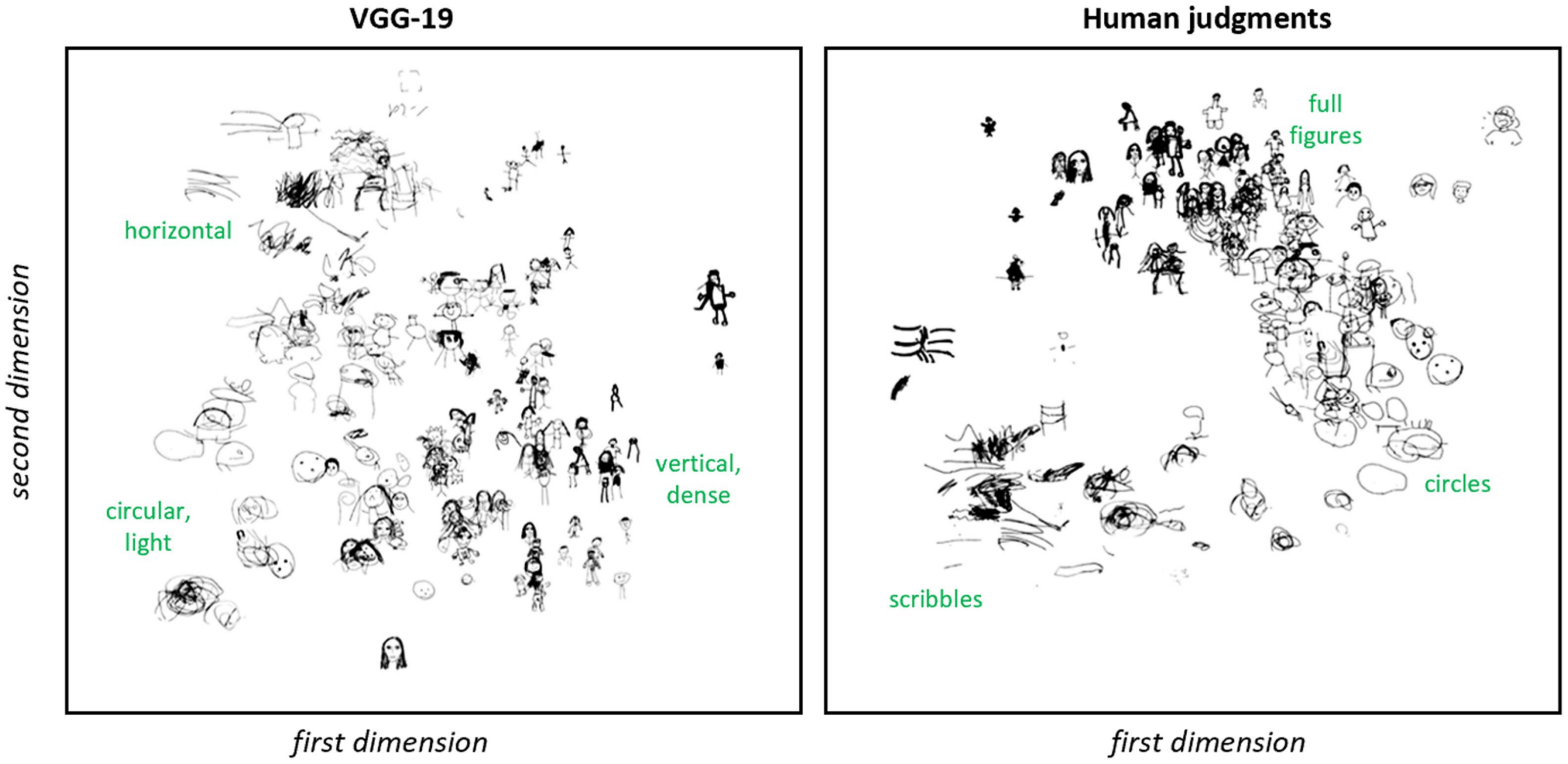
Two-dimensional embeddings of drawings. Two-dimensional embeddings for a subset of human figure drawings based on VGG-19 vectors (left) vs. human judgments of similarity (right). By inspection, each technique captures some aspects of structure. For VGG-19, circular shapes composed of light strokes are grouped in the bottom left, images with many horizontal strokes appear near the top, and drawings with dense strokes oriented vertically appear toward the right. For human judgments, sketches trace out a manifold reminiscent of a common developmental trajectory, with scribbles in the bottom left transitioning to circles toward the right and then to fuller depictions of the whole figure toward the top middle.

Before tackling the questions laid out in the introduction, it is useful to get an initial qualitative sense of the structure expressed by machine-and human-derived latent feature vectors, and to evaluate whether the two approaches capture distinct information about the similarity relations among drawings. We began by plotting the 2D embeddings for a subset of drawings as shown in Figure 5. By visual inspection, the two approaches each capture discernible but somewhat different structure across drawings. Embeddings from human judgments lie along a curve in the 2D embedding space, with an ordering that appears to reflect the developmental progression from scribbles to circles to fully differentiated body parts. This organization is more difficult to see in the VGG-19 embeddings, which nevertheless clearly capture some elements of similarity amongst the drawings. For instance, by visual inspection, the VGG-19 embeddings appear to group together larger circular drawings composed of light strokes, including both circular faces and scribbles. These can be seen in the lower left of the figure. Larger drawings with many light horizontal strokes, including a human figure with arms outstretched and horizontal scribbles are grouped in the top left of the figure. Images that are smaller in relative height and width on the drawing surface, with dense strokes and a vertical orientation, appear clustered toward the right middle of the figure. Note that human embeddings group together round and horizontal scribbles that are widely separated in the VGG-19 plot, while VGG-19 embeddings group tadpoles and fully-differentiated figures when these are similar in size, vertical orientation and stroke weight.

**Figure 6:**
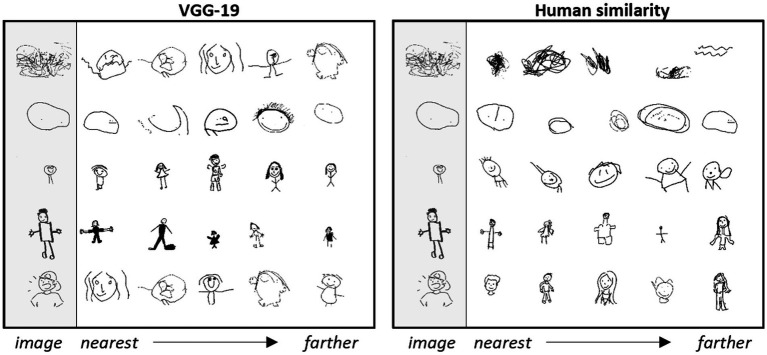
K-nearest neighbors of five-dimensional embedding spaces. Nearest neighbors for five reference items in the 5D embedding space for machine- (left) or human- (right) derived latent feature vectors. Rows show the five closest neighbors to the reference item (gray column) in order of proximity. The same reference items are used in each space, but the two spaces capture different neighborhood relations.

To understand whether the apparent differences between machine-and human-derived embeddings are an artifact of compression to just two dimensions, we also considered 5D embeddings generated from both human judgments and VGG-19 representations. We first visually inspected the five nearest neighbors in each 5D space for a set of reference images. Figure 6 shows a representative set of images. The nearest neighbors are completely non-overlapping in the two spaces, suggesting that they capture different similarity relations. The human-derived embeddings again appear to capture the developmental “stage” of the participants: the scribble in the top left is near other images that fit within the category of “scribbles”; circle-faces are near other circle-faces; full figures are near other full-figures; etc. In contrast, the same scribble is near images recognizable as human figures in the machine embeddings; the tadpole in the middle is near fully-articulated figures; and the well-rendered figure in the bottom left is near drawings highly variable in quality.

To test whether the differences arising in this small set of sample images are more broadly characteristic of the two spaces, we computed, for each image, how often the nearest neighbor in one embedding space appeared in the *top ten closest items* for the same referent in the other space. In both cases, for over 90% of the images, the nearest neighbor in one space was *not* among the top ten nearest images in the other. Thus even in this broader space, the embeddings capture different similarity relations amongst the images.

Finally, we used regression to quantify how similar the 2D machine-and human-derived embedding spaces are to one another. Each of the two embeddings situates drawings in two dimensions, so we fit and evaluated four regression models, each using one embedding dimension in a given space as the dependent variable. All four models accounted for significant variance in the dependent measure, showing that the two spaces are not completely independent (see Table 1). Yet neither are they identical: in all four regressions, more than half the variance in a drawing’s location along one dimension in a given space remains unexplained by its joint coordinates in the other space. Thus the machine-and human-derived embeddings, though not completely independent, nevertheless express quite different structure amongst the drawings.

**Table 1:** Regressions predicting coordinates of embedding in one space from those in the other.

Model	*R*^2^	*p*
X_human_ = X_machine_ * Y_machine_	0.21	<0.001
Y_human_ = X_machine_ * Y_machine_	0.46	<0.001
X_machine_ = X_human_ * Y_human_	0.48	<0.001
Y_machine_ = X_human_ * Y_human_	0.23	<0.001

Each model predicts the coordinates of drawings along a given dimension in the human- or machine-derived embedding from both coordinates and their interaction in the other space. X and Y indicate the first and second dimensions of the human- or machine-derived embedding.

Note that, whereas the human embeddings appear to capture structure expressing the developmental trajectory of human-figure drawing, it is less clear what information governs the structure of the machine embeddings. While our qualitative observations hint at some possible characteristics that the network may be exploiting (e.g., stroke density, size of the drawings on the surface, vertical/horizontalness, etc.), these may or may not correctly reflect the information guiding the model representations. One potential advantage of using neural network feature vectors to characterize the structure of drawings is that, precisely because of their opacity, such models may discern structure beyond what the human eye naturally detects that is difficult to analytically extract through simpler means. The analyses in this section demonstrate that machine-based representations capture similarity relations that are quite different from those governing human perceptual judgments—so regardless of the driving image characteristics, it is an empirical question whether the machine-perceived structure captures cognitive, motor, or behavioral characteristics of the participant.

**Figure 7:**
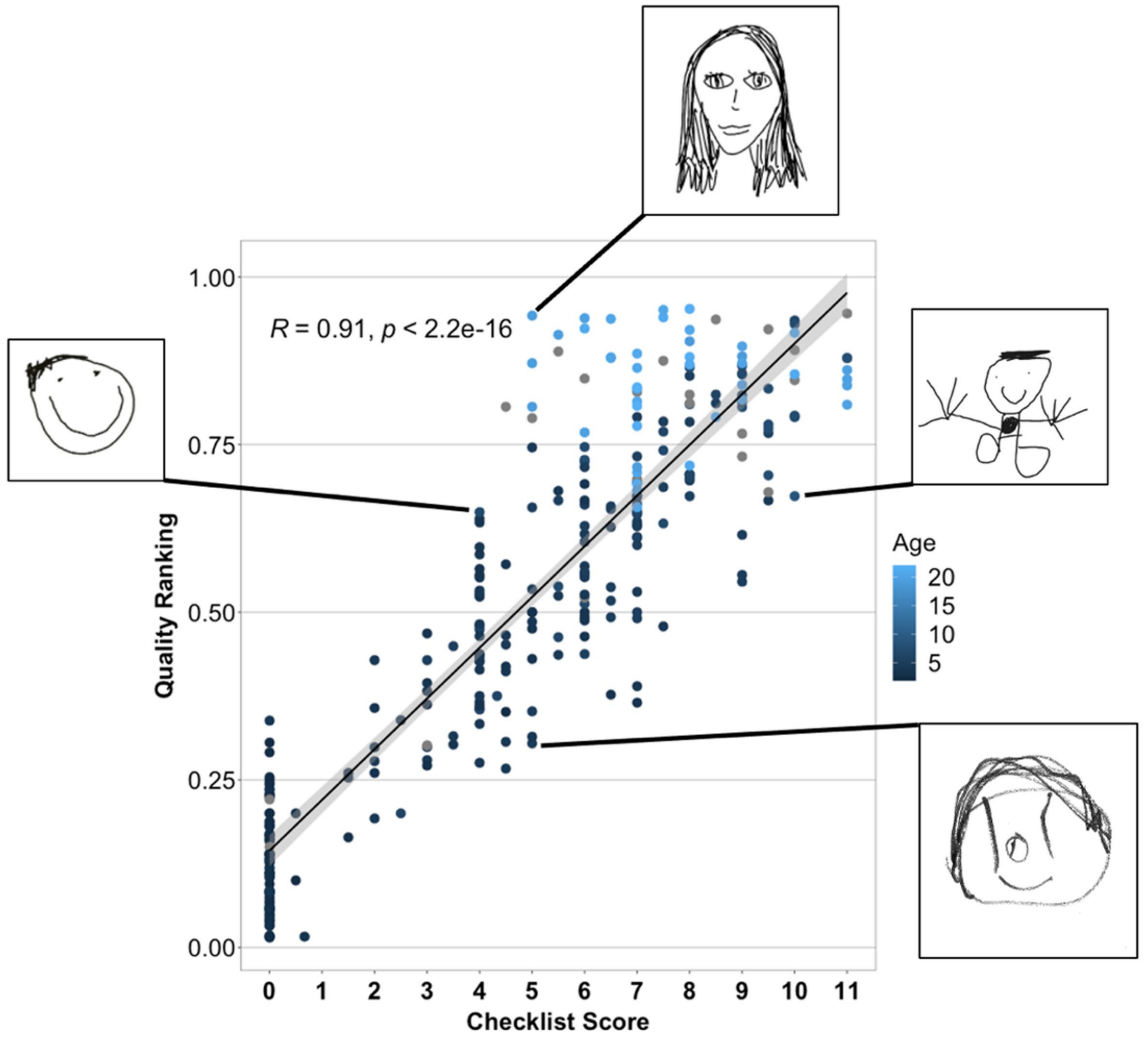
Relation between the crowd-sourced quality-ranking and the Draw-A-Child checklist score. The top and bottom callouts show drawings that received the same checklist score but differ in quality-rank, while the two middle callouts show drawings that received similar quality-rank scores but different checklist scores.

We next considered how the crowd-sourced quality-rank score relates to the conventional Draw-A-Child 12-point checklist score. Recall that the quality-rank score is based on many non-expert forced-choice evaluations of comparative drawing quality, while the checklist score is based on evaluating the presence of 12 key features by trained raters. Nevertheless, the two metrics were highly correlated [*r*(303) = 0.91, *p* < 0.001, CI (0.88–0.92); see Figure 7], though items receiving the same checklist score varied nontrivially in their quality score and vice versa. Figure 7 shows some examples in callouts: two drawings both receiving a checklist score of 5 clearly differ in drawing quality, while two drawings receiving a quality-rank score near 0.65 appear to be of similar quality but differ in the parts included. Thus, the checklist and quality-rank, despite their high correlation, capture somewhat different information about each rendering. If the features appearing in the checklist are especially important for understanding aspects of development beyond just capturing image quality, the checklist metric should better predict individual variability on those aspects than does the quality-rank score. If, however, the main utility of the checklist for understanding some component of cognition is to capture overall drawing quality, the quality-rank metric should account for as much or more variance on that component as does the checklist score.

To better characterize the extent to which the new metrics express structure similar or different to that captured by the checklist score we tested these relationships in two ways. In the first analysis, we fit regression models to predict a drawing’s checklist score (averaged across the two raters) from each of the new metrics (quality-rank score, human-derived embedding coordinates, and machine-derived embedding coordinates) taken independently and in combination. The results are shown in Table 2. Both human-derived measures independently accounted for over 80% of the variance in checklist scores, and in combination they accounted for significantly more variance than either considered alone (86%, *p* < 0.0001 in contrast against best independent model). Machine-derived embeddings accounted for just 36% of the variation in checklist scores when considered independently, though this rose to 84% when quality rank and its interactions were added to the model. When all new metrics and their interactions were added to the model, model fit rose significantly, accounting for 89% of the variance in checklist scores (*p* < 0.0001 for fit contrast to next-best model). Thus the bulk of the information captured by the checklist score is also expressed jointly by the new metrics.

**Table 2:** Adjusted *r*^2^ and model-comparison *p* values for regressions predicting a drawing’s checklist score from the new metrics.

Predictors	Adj. *r*^2^	Contrast to alternative H
Quality Ranking (QR) only	0.82	*p* < 0.0001 vs. null
Human Embedding (HE) only	0.84	*p* < 0.0001 vs. null
Machine Embedding (ME) only	0.36	*p* < 0.0001 vs. null
QR * Human embedding	0.87	*p* < 0.0001 vs. HE only
QR * Machine embedding	0.84	*p* < 0.0001 vs. QR only
QR * HE * ME	0.89	*p* < 0.0001 vs. QR * HE

In the second analysis we assessed whether human- and machine-derived estimates of pairwise similarities amongst drawings (expressed as distances between points in the corresponding embeddings) capture structure distinct from the similarities in their checklist scores. For each embedding space and for the checklist scores, we computed the Euclidean distances between all drawing pairs. For the checklist, each entry was simply the absolute value of the difference in checklist scores. Across all unique pairs, we then computed the correlations of these distances for each pair of metrics. For the checklist and human-derived embedding distances, the correlation was *r* = 0.70, suggesting that the two measures capture related but non-identical information (49% shared variance) about similarities amongst drawings. The correlation between human- and machine-derived embedding distances was smaller (*r* = 0.31, 10% shared variance) and between checklist and machine-derived embedding distances smaller still (*r* = 0.22, 4% shared variance). Although it is evident that each metric captures distinct information about similarities existing amongst the various drawings, exactly where and how those differences arise is less clear. One way to explore what may underlie both differences and similarities that define these metrics is to consider the predictive ability of each approach on measured attributes of the participants that produced the drawings.

### Predicting demographic characteristics of the participants

The preceding exploratory analyses show that the new metrics each express aspects of structure in drawing different from that captured by the standard scoring metric, and different from each other. The next question is whether these varieties of structure in turn reliably capture information about the participant. As an initial proof of concept, we first considered the participant’s age and gender, focusing on these demographic factors for several reasons. First, they represent two different prediction problems of key interest for assessment, that is, prediction of a continuous (age) and a categorical (gender) dependent measure. Second, age and gender data are available for all study participants, providing good power for a proof of concept analysis. Third, by analyzing the fits of different predictive models we can assess whether the three new metrics capture any information beyond that already expressed in checklists, and whether they capture similar or different components of variation in these dependent measures. Fourth, some prior work has suggested that DAP-style tests may show reliable sex differences, with girls generally producing more detailed drawings at an earlier age than boys (Goodenough, 1926; Goodenough and Harris, 1950; Harris, 1963; Naglieri, 1988; Cox, 1993; Lange-Küttner et al., 2002). Finally, the demonstration on these simple demographic characteristics provides a blueprint for the subsequent analyses.

In all analyses, age data were log-transformed to better approximate a normal distribution, while gender data were coded as a discrete binary factor. Models predicting age were fit using linear least-squares regression and evaluated using the *r*^2^ metric, while those predicting gender were fit using logistic regression and evaluated using the area under the ROC curve (AUC or area-under-curve) estimated on held-out items from the fitted classifier. For these latter assessments, models were fit to 90% of the data and AUC was computed for the remaining 10%, held out at random. This procedure was run 100 times, with a different set of random hold-outs each time, and model performance was taken as the mean estimated AUC across these folds.

Otherwise, prediction of age and gender followed the same stepwise procedure. Step 1 first fit a “baseline” model predicting the dependent measure from the checklist score and including the other sociodemographic factor as a covariate of no interest. To assess whether the quality-ranking score carries information beyond that captured by the checklist score, a second model was fit including both checklist and quality-rank and their interaction. Any resulting change in model fit was evaluated using ANOVA. Step 2 then evaluated whether the addition of image coordinates from the machine-and human-derived 2D embeddings, considered separately, reliably improved model fit relative to the best-performing model of Step 1. We focused on 2D embeddings rather than higher-dimensional embeddings simply due to power considerations: since the number of terms in the regression increases exponentially with the number of predictors (when interactions are included), and given the size of our dataset and the required covariates in each model, two additional predictors beyond age, gender, and checklist/quality-rank were the most we could include. There is no principled reason, however, why higher-dimensional embeddings could not be included for analyses of larger datasets. Finally, Step 3 evaluated whether machine-and human-derived coordinates account for unique variance in the dependent measure by adding embedding data from both methods and comparing change in model fit to the best-performing model from Step 2.

The results are shown in Table 3. The checklist score predicted 49% of the variance in log age, but this increased to 64% when checklist score was replaced with the quality-rank score, when both models covaried out gender. A comparison of the model with both metrics to the model with checklist alone showed that quality-rank accounted for significant additional variance beyond that explained by the checklist score. Further, adding either the human- or the machine-derived embedding coordinates significantly improved model fit, and by an equal amount, with both models showing an adjusted *r*^2^ of 0.69. Including both human- and machine-derived embeddings did not reliably improve fit compared to either of these alone, *r*^2^ = 0.69, suggesting that both embeddings capture the same additional variation after taking quality-rank into account.

**Table 3:** Model fits predicting demographic characteristics of participants.

Dependent variable	Metric	*n*	Baseline	Quality ranking	Human embedding	Machine embedding	Both embeddings
Age	Adj. *r*^2^	280	0.49*** < 0.64***		0.69***	0.69***	0.69
Gender	AUC	287	0.62*** < 0.67***		0.74*	0.74*	0.87***
*Comparison model:*			Null	Null	QR	QR	QR * HE

Age models include gender and its interactions with other variables as regressors of no interest, and vice versa for Gender models. The Baseline model includes checklist only, while the Quality-Ranking models replace this with the quality-rank (QR) score. Significance tests for these are against the null hypothesis, while the comparison signs (greater/less than) indicate whether one metric accounts for reliably more/less variance than another. Asterisks indicate significance levels at *p < 0.05, **p < 0.01, ***p < 0.001.

Next, the baseline model showed reliable above-chance classification of the participants gender, with higher scores on the checklist measure predicting a greater likelihood that the drawing was made by a female participant after covarying out effects of age (see Table 3), consistent with prior work (Goodenough, 1926; Goodenough and Harris, 1950; Harris, 1963; Naglieri, 1988; Cox, 1993; Lange-Küttner et al., 2002). This predictive accuracy again improved reliably when the checklist score was replaced with the quality-rank score. Both human- and machine-derived embedding coordinates significantly improved model’s predictive accuracy compared to the quality-rank alone, and the incorporation of both embeddings together produced significantly better classification accuracy compared to either alone. The model including all three metrics (and including age as a covariate of no interest) showed a remarkable AUC value of 0.87—that is, 87% accuracy discriminating males from females solely based on overall quality and latent structure in the drawings. Interestingly, the relationship between the drawing quality-rank score and the probability of being female remained positive in all models—suggesting, again consistent with prior work, that girls produce drawings perceived as higher quality than those produced by boys, even taking other aspects of drawing structure into account.

### Predicting motor and cognitive characteristics of the participants

Finally, we evaluated whether the new metrics carry information about aspects of cognition and behavior measured by the ASQ and about motor abilities as measured through both the parental report within the ASQ, and the practical measures of pinch and grip strength. As already noted, these measures were collected for only a subset of child participants, yielding a total of 109–115 drawings from participants whose parents contributed ASQ responses, and 198 from participants who completed the pinch/grip measures. We also note that the ASQ typically serves as a screening measure for which most children will perform near ceiling, yielding comparatively little variance and a corresponding lack of power for regression. Nevertheless, the inclusion of these measures is useful for several reasons. First, should reliable effects be observed despite the narrow variance, this provides strong evidence that latent structure of drawings can contain information useful for developing a cognitive/behavioral profile of the developing child. Second, the ASQ includes subscales assessing different aspects of behavior, allowing us to determine whether latent structure in drawings carries more information about some components than others. Third, the comparison of fits for models with new metrics to models including just the checklist score allows us to evaluate whether the new metrics carry information beyond that already captured by standard checklist measures. Fourth, the comparison of models with human- versus machine-derived embeddings allows us to evaluate whether the structure captured by these techniques express similar or different aspects of the child’s cognitive, motor, and behavioral makeup.

Our analysis followed the same stepwise plan from the demographic study, with three minor changes. First, age was not log-transformed since participants were all children and age was approximately normally distributed; similar results were obtained with log-transformed age data. Second, all models included age, gender, and their interactions as covariates of no interest. Third, we did not complete step 3 of the regression in which both human- and machine-derived embeddings and their interactions were added jointly to all other terms, since these models had a very large number of parameters relative to the number of data points. Otherwise the comparison of baseline and quality-rank models, and the further addition of embedding coordinates from human- and machine-derived data, proceeded as described for the demographic analysis.

The results are shown in Table 4. Several observations are of interest. First, for three ASQ subscales (Communication, Gross Motor, and Fine Motor), the standard checklist score accounted for significantly more variance than did the quality-rank score, in contrast to our analysis of demographic factors (*adj r^2^* values of 0.04, 0.27, and 0.33, respectively). This suggests that checklists may indeed capture some important information beyond overall image quality, especially as regards parental evaluations of the child’s motor abilities. For the remaining subscales and for the ASQ total score the two measures captured comparable variation. Second, the addition of embedding coordinates to predictive models significantly improved model fit for 5 of the 8 measures, including some measures clearly relevant to drawing (Grip, Pinch, and ASQ Fine Motor) but also some measures with no transparent relationship to drawing (the Communication and Personal/Social subscales of the ASQ). It is worth noting the large amount of variance explained by all drawing measures for both Grip and Pinch strength. This finding underscores the interrelationship between the structure of the drawing and the child’s physical abilities. Third, in all five cases this additional variance was captured by the machine-derived embeddings; in only one case (ASQ Fine Motor) was the additional variance also captured by the human-derived embeddings. Fourth and finally, where embedding coordinates helped prediction, the models captured a remarkable amount of variance—between 41 and 74%—in the dependent measure.

**Table 4:** Model fits predicting cognitive/behavioral and motor characteristics of participants.

Dependent variable	*n*	Baseline	Quality ranking	Human embedding	Machine embedding
ASQ total	115	0.12** = 0.07*		0.15	0.15
ASQ communication	115	0.04* > 0.00		0.04	*adj* = 0.18*
					*mult* = 0.41*
ASQ gross motor	115	0.27*** > 0.19***		0.25	0.26
ASQ fine motor	115	0.33*** > 0.27***		*adj = 0*.43*	*adj* = 0.49**
				*mult* = 0.59*	*mult* = 0.63**
ASQ problem solving	109	0.05 = 0.07*		0.00	0.02
ASQ personal/social	109	0.33*** = 0.30***		0.40	*adj = 0*.48**
					*mult* = 0.63**
Pinch	198	0.56*** = 0.54***		0.57	*adj =* 0.63***
					*mult = 0*.69***
Grip	198	0.65*** = 0.66***		0.68	*adj =* 0.70**
					*mult =* 0.74**
*Comparison model:*		Null	Null	Model with higher *r*^2^ Baseline or QR	

Values are adjusted *r*^2^; multiple *r*^2^ is also reported for models where embedding coordinates significantly improve model fit. All models include age and gender and their interactions as covariates of no interest. Baseline models additionally include the checklist score while Quality Ranking models replace this with quality-rank (QR) score. All models include all interactions. For baseline and QR, significance tests are against the null hypothesis while equal/greater-than signs indicate whether either metric accounts for significantly more variance than the other. Models with embedding coordinates were evaluated against the better-fitting model in the comparison of baseline and QR. Asterisks indicate statistical significance at *p < 0.05, **p < 0.01, ***p < 0.001.

## Discussion

A long tradition of research has endeavored to use children’s drawings of the human figure to better understand their cognitive and behavioral development. A key challenge has been to develop methods for quantifying the structure that appears in such drawings. We introduced three new metrics derived from recent innovations in machine vision and crowd-sourcing of human judgments, and showed that these capture a wealth of information about the participant beyond that expressed by standard measures, including age, gender, motor abilities, personal/social behaviors, and communicative skills. Machine-and human-derived metrics captured somewhat different aspects of structure across drawings, and each were independently useful for predicting some participant characteristics; however, only the machine-derived metrics explained significant additional variation in the motor and ASQ subscales. Since the human embeddings reflect perceived similarity, this difference must arise because the neural network representations capture informative structure that is non-obvious to human perceivers (or at least does not prominently drive human similarity judgments) and is related to characteristics measured by the ASQ subscales and motor tasks. The contrast shows why the machine representations are useful over and above metrics based solely on structure that people readily perceive: they may express varieties of structure that do not occur to human raters.

A central goal of this work was simply to evaluate whether it is possible to mine information from children’s drawings relevant to understanding their cognitive, motor, and behavioral makeup, in ways that go beyond standard checklist measures. The question is important because of the special role that drawing can potentially play in developmental assessment. Where most assessment tools require children to perform unfamiliar and potentially unmotivating tasks encountered only during the assessment itself, drawing is a common activity that most children enjoy and pursue in daily life. Like language, drawing requires coordination of many faculties typically studied independently, including perception, conceptual knowledge, planning, sequencing, and motor control—yet because it relies minimally on language competency, it provides a means of understanding interactions amongst these abilities independent of linguistic skill. Our positive results suggest that drawings carry information far beyond that recognized in prior work, paving the way for more comprehensive use of drawings in future evaluative work.

Beyond this proof of concept, the current results also suggest some specific relationships between qualities of drawings and characteristics of participants. Perhaps most obviously, the new metrics predicted aspects of motor control with remarkable accuracy, including pinch and grip strength as well as the ASQ Fine Motor subscale. Though it seems clear that motor abilities should influence drawing, the use of drawing as an assessment has primarily focused on other factors such as intelligence (Goodenough, 1926; Harris, 1963; Naglieri, 1988), personality (Machover, 1949; Hammer, 1958), and social/emotional disturbance (Koppitz, 1968; Naglieri et al., 1991). Indeed, assessments of supposed “higher order” aspects of cognition often ignore or downplay potential contributions of motor function to the measured behavior. The current results show that the same behavior known from prior work to predict intelligence can also predict significant variance in motor function, raising the possibility that these are not independent but linked. Future work with larger samples and richer measures is needed to assess, for instance, whether latent structure in drawings predicts different characteristics independently, or whether motor functioning mediates predictive relationships with intelligence and other measures (or vice versa).

The new metrics also reliably predicted variation on two ASQ subscales not transparently related to drawing, specifically those for Communication and Personal/Social development. It is possible that these relationships result from the reliance of the associated subscales on motor function. For example, children demonstrate reciprocal communication skills in the ASQ by correctly moving a book after a verbal request, or by successfully using a zipper based on demonstration and instruction. Likewise, tasks that are recorded as personal/social achievements in the ASQ include the use of a spoon and fork when eating, unscrewing a lid from a jar, and by copying behaviors children have witnessed like drinking from a glass or combing one’s own hair. It may be that motor functioning revealed by characteristics of drawings likewise influence behavior on these measures—an important possibility since research on human figure drawing often views drawings as providing a window into the mind without regard for the physical demands of the task itself. Alternatively, the predictive relationship with Communication and Personal/Social subscales may reflect other characteristics of the child not mediated by motor function. For instance, since the task requires rendering of a human figure, it may reflect differences in the child’s interest, ability or experience interacting with others—factors that may lead to better or just different renderings when the child is asked to draw another person. Again, further work with larger and more diverse samples and richer metrics can adjudicate these possibilities.

The pattern of female participants scoring higher on checklist-based measures of human figure drawing, which Goodenough (1926) noted in the original Draw-A-Man scale, was replicated in our sample on the 12-item TEDS adaptation of the Draw-A-Child checklist. However, our new metric of drawing quality better predicted participant gender compared to the checklist, while inclusion of both machine-and human-derived latent feature vectors further boosted predictive accuracy. In all models, better drawing scores—whether checklist or quality-rank—predicted a larger probability that the participant was female. This phenomenon, and the remarkable predictive accuracy of models that incorporate human- or machine-based latent features, may reflect the Draw-A-Child test’s instruction for participants to draw a child of the same gender, coupled with cultural norms about how gender is depicted. Western conventions often depict girls as having long hair and triangular bodies to denote dresses. Presence of hair and clothing constitute two items on the TEDS variant of the drawing checklist, potentially leading to higher scores for girls on this basis—though it is worth noting that girls still score higher in studies that attempt to control for such confounds, for instance by asking that female figures be depicted in a swimsuit (Lange-Küttner et al., 2002). Likewise, it may be that drawings possessing these or other culturally gendered details are judged to be higher in quality than those that do not, influencing the quality-rank score; and that the tendency to share such features impacts the organization of drawings in both human- and machine-derived embeddings, explaining their contribution to gender prediction. Alternatively, it may be that the gender phenomenon represents something more intrinsic to a child’s cognitive, social, or motor makeup, beyond just the differences in how boys and girls are conventionally depicted. Future work could address this question by applying comparable techniques to other kinds of drawings that are not intrinsically gendered, such as 3D shapes (Lange-Küttner, 2000; Lange-Küttner and Ebersbach, 2013).

Our novel measures did not reliably predict the overall ASQ-total score, nor scores on Gross Motor and Problem Solving subscales. As the ASQ is primarily a measure of developmental delays and our sample included only typically developing children, it is perhaps not surprising that our measures did not predict the ASQ total score, a general indicator of developmental delay. Though Kirkorian et al. (2020) did not conduct a formal assessment beyond the ASQ-3 for developmental delays, none of the parents reported that their children had any diagnosed conditions. The absence of reliable prediction for the Gross Motor subscale is more interesting, as it suggests that latent structure in drawings may not characterize overall motor ability generally, but may be more informative about aspects of fine manual motor control required for drawing. With regard to Problem Solving, the null result may arise for either of two reasons. First, this subscale more than any other in the ASQ measures aspects of development not directly related to drawing (e.g., verbal skills, including color identification, counting as well as pretend play). Second, this subscale showed the least variation in our sample, with 73% of respondents receiving the maximal possible score—thus the null result may reflect a large number of ceiling responders.

One limitation of the current research is that it is not entirely clear what features of the human figure drawings influence the new human- and machine-derived metrics. The regression results show that each approach can capture distinct information from one another and from the checklist score—for instance, the quality-rank score explains more variation than the checklist in age (*adj r^2^* = 0.64 vs. *adj r^2^* = 0.49) and gender (*adj r^2^* = 0.67 vs. *adj r^2^* = 0.62), but less in the ASQ motor subscales (see Table 2); and, while human-derived embeddings did not predict variation beyond machine-derived embeddings for any measure, nevertheless the two spaces identified measurably different structure amongst the various drawings as evidenced by the regression fits in Table 1: regression models predicting coordinates of a drawing in one space from those in the other accounted for less than half the variance on each dimension, both predicting machine-derived embeddings from human-derived embeddings and vice versa. Understanding precisely what kinds of structure each measure captures, and connecting these to features and characteristics expressed by checklist measures, will facilitate integration of this new work with the long history and rich literature on children’s drawings. Nevertheless, the current results suggest that our novel metrics have some diagnostic specificity relevant for characterizing different aspects of cognition and behavior even in healthy, typically-developing populations.

## Final thoughts and future directions

Given the commonality and enjoyment of children sitting down to draw, it is not surprising that there is a long history of curiosity about what a drawing can tell us about a child’s inner life. Our results suggest that human figure drawings are not a direct window into the child’s mind, but are best viewed as artifacts that reflect the joint operation of many different factors, including perceptual and cognitive skills, motor factors, and possibly social and communicative abilities. In the current study we found that both machine-learning and human-similarity judgements could be used to capture underlying structure related to each of these participant characteristics, even among typically-developing children and using screening metrics that limit individual variation. Prior research incorporating devices such as pressure sensitive tablets has demonstrated that both age and the task demands can impact both the pressure applied when drawing but also the number of pauses and line breaks within a shape or figure (Lange-Küttner, 1998, 2000; Tabatabaey-Mashadi et al., 2015). Our results suggest that computational methodologies, especially machine vision, may likewise provide a useful path to identify how related features like stroke density and smoothness of the contour of a drawing may serve as indicators of participant attributes that lie outside of checklist-based measures. Of note, the sample of images used in this study were not collected using technology to monitor the pressure applied by participants when drawing, and so our novel approaches may provide an alternative solution to consider the physical nature of drawing within image collections that were not collected using such media. A key goal for future research will be to assess whether similar metrics, collected in a larger and more diverse sample, and using richer cognitive/behavioral measures, can reshape our understanding of typical and typical patterns of development.

## Data availability statement

The raw data supporting the conclusions of this article will be made available by the authors, without undue reservation.

## Ethics statement

The studies involving human participants were reviewed and approved by Institutional Review Board for Education and Social/Behavioral Science at the University of Wisconsin-Madison. Written informed consent to participate in this study was provided by the participants’ legal guardian/next of kin.

## Author contributions

DS was involved in related work. HK, BT, and KR collected the initial data and contributed to the manuscript revision, TR contributed to both statistical analysis and to the framing of the original manuscript. All authors approved the submitted version.

## Conflict of interest

The authors declare that the research was conducted in the absence of any commercial or financial relationships that could be construed as a potential conflict of interest.

## Publisher’s note

All claims expressed in this article are solely those of the authors and do not necessarily represent those of their affiliated organizations, or those of the publisher, the editors and the reviewers. Any product that may be evaluated in this article, or claim that may be made by its manufacturer, is not guaranteed or endorsed by the publisher.
